# Supplementary figures and images for: Dynamic Alteration Profile and New Role of RNA m6A Methylation in Replicative and H2O2-Induced Premature Senescence of Human Embryonic Lung Fibroblasts
Source: Int J Mol Sci. 2022 Aug 17;23(16):9271. doi: 10.3390/ijms23169271 (PMC9408987; doi:10.3390/ijms23169271)

Supplementary Figure S1

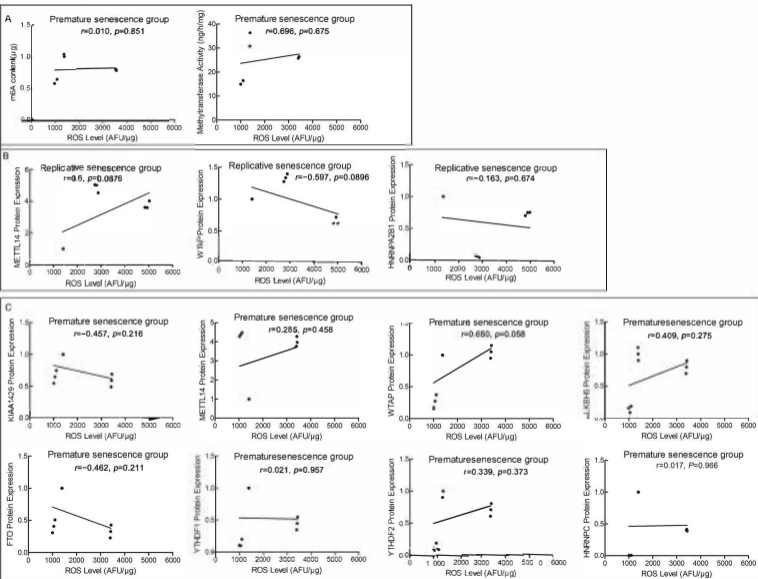

Supplement: Supplementary file 1 [file ijms-23-09271-s001.zip › ijms-1834816-supplementary.pdf]
